# Safety Risk Estimation of Construction Project Based on Energy Transfer Model and System Dynamics: A Case Study of Collapse Accident in China

**DOI:** 10.3390/ijerph192114386

**Published:** 2022-11-03

**Authors:** Yongcheng Zhang, Xuejiao Xing, Maxwell Fordjour Antwi-Afari, Mingqing Wu

**Affiliations:** 1 School of Management Engineering, Huaiyin Institute of Technology, Huai’an 223003, China; 2 School of Finance, Zhongnan University of Economics and Law, Wuhan 430074, China; 3 School of Civil and Hydraulic Engineering, Huazhong University of Science and Technology, Wuhan 430074, China; 4 Department of Civil Engineering, College of Engineering and Physical Sciences, Aston University, Birmingham B4 7ET, UK; 5 School of Economics and Management, Nanjing Tech University, Nanjing 211816, China

**Keywords:** safety risk estimation, construction project, collapse accident, energy transfer model, system dynamics

## Abstract

Analyzing and understanding the occurrence and evolution mechanisms of construction accidents are important for construction safety management. This study proposed a hybrid approach of integrating the energy transfer model (ETM) and system dynamics (SD) theory to delineate the entire evolution stage of the construction accident. Specifically, the Fengcheng Power Plant construction platform collapse accident (FPCA) was taken as a practical case study. First, the ETM is applied to demonstrate the evolving nature of the accident. Then, the network of the accident-causing factors is constructed using the SD theory to analyze the dynamic change characteristics. The results indicate that the accident was caused by risk factors with complex interactions at the management level. An energy constraint failure occurred when the transfer of dangerous energy transpired at the physical entity level, inducing the event. The proposed approach can provide a useful reference for safety risk estimation and management in future major construction projects.

## 1. Introduction

The construction industry is characterized by a large scale of accidents that occur with high probability, wide involvement, and are associated with diverse hazard sources [1]. Therefore, the construction industry is one of the most dangerous fields. According to statistics from the United States, United Kingdom, South Korea, and other countries, the death rate of injuries at construction sites is the highest among all other industries [2]. According to the statistics from the Ministry of Housing and Urban-Rural Development, 1426 accidents, such as foundation pit collapses, elevator falls, and tower crane collapses, have occurred in China over the past two years. Many deaths and injuries have been reported in the construction industry [3]. For example, six people died and six were injured in a collapse accident of a steel structure at the Hupanli project in the Jinhua development zone, Zhejiang province, on 23 November 2021. During the civil engineering construction of the Chengdu transit line 17 in Sichuan province, parts of sheds for dust and noise reduction collapsed, thereby causing 4 deaths and 14 injured persons on 10 September 2021.

Given these reasons, scholars have conducted studies on risk management and accidents analysis based on some models or technologies in engineering construction (e.g., deep learning algorithms [4,5], the system dynamics (SD) method [6], and building information modeling (BIM) [7]), which were summarized in Table 1. For example, Zhong et al. extracted and classified accident contents automatically by using a convolutional neural network (CNN) model and a potential Dirichlet distribution model, and found the relationship between causal variables, in order to better understand the processes leading to accidents [8]. Zhou et al. used the network theory and energy changes model to discuss the complexity of subway construction accident networks [9]. Kim et al. proposed a BIM-based hazard recognition and evaluation methodology for automating the construction site risk assessment [7].

Based on previous studies, it is important to understand the potential risk factors for preventing construction safety accidents. However, little research has focused on delineating the entire evolution stage of the accident, which is of vital importance for the risk prevention of similar accidents [15].

Bird et al. proposed the accident tendency theory and domino theory, respectively [16]. On this basis, they have also proposed the linear causality model, also known as the Swiss cheese model. With the development of high-risk social-technical systems, Rasmussen’s social-technical causality model adopted a broad system perspective to identify the causal factors of various individuals and organizations in a complex system [17]. From the perspective of physics, different forms of energy conversion are completed by doing work. The theory of energy transfer posits that safety accidents represent abnormal energy transfer instances that may lead to accident-related injuries, and accidents are often the result of poor energy control [13]. As the carrier of the accident, energy determines the severity of the accident, and the failure of the energy constraint determines the probability of the accident as a necessary condition. Specifically, the energy transfer model (ETM) interprets gradual processes that lead to changes in the transfer of dangerous energy from the perspective of micro-level energy, analyzes the failure factors of the accident constraints, and grasps the internal essential characteristics of the accident [9]. The fundamental factors that lead to accident understanding are better understood through the analysis of the energy transfer changes in the accident.

The system dynamics (SD) framework was proposed to conduct qualitative and quantitative analyses of complex systems with nonlinear, delayed, and multiple feedback controls, and describes causal relationships among involved factors [18]. Seresht and Fayek-integrated fuzzy SD and hybrid neuro-fuzzy systems, and applied them to define nonlinear, complex, and high-dimensional relations among system variables for more accurate architectural applications [19]. Yu et al. constructed a SD model of the unsafe behavior of coal miners, reordered the influencing factors, proposed joint intervention strategies for safety management, individual factors, group factors, natural environment, and other aspects, and carried out simulations [16]. The SD model is a qualitative and quantitative method for analyzing complex systems, which can capture causal loops, and can explain network interactions that lead to accidents [16,18,19]. From the perspective of macro-management, the SD model can be used for analyzing multiple and complex network relationships among the participants of the engineering project management. Characteristics of external factors for the occurrence of accidents can be explained. Therefore, this paper presented a novel approach to delineate the entire evolution stage of the construction accident and estimate the involved safety risks, based on the ETM and SD. From the perspectives of technology and management, the construction safety risk can be estimated with the complicated causal mechanisms analyzed. In particular, a collapse accident in China, the Fengcheng Power Plant construction platform collapse accident (FPCA), was chosen as a case study of this research. It was a particularly serious accident in industrial construction involving different participants, several industries, causes, and multilevel contracting [20]. Considering the complexity of risk factors of this accident and the deficiency of general linear causal models, systematic thinking was strengthened based on traditional accident analysis [2]. All complexities under the multilinear accident chain integration have been understood from the perspective of dangerous energy transfer and inadequate management [20]. The proposed approach is expected to fully explore the potential causal relationships leading to accidents, and understand the formation mechanisms and occurrence process of accidents. In this way, novel ideas and methods for the safe estimation and management of major construction projects can be supported.

## 2. Materials and Methods

### 2.1. Context

**Figure 1:**
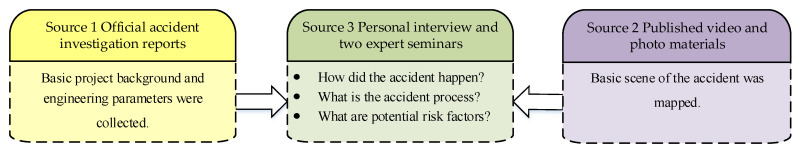
Data collection process of the FPCA for a case study.

The project context of the FPCA was gathered from multiple sources (Figure 1). The basic project background and engineering parameters were collected from official accident investigation reports. Published video and photo materials related to the FPCA were collected at the same time to map the scene of the accident. Specifically, personal interviews and two expert seminars were conducted by inviting six experienced domain experts, involving three civil engineering scholars and three engineering practitioners from Jiangsu projects. During the seminars, the accident process and involved potential risks were stated and discussed. Then, the case study on FPCA was carried out based on the organized accident information materials.

**Figure 2:**
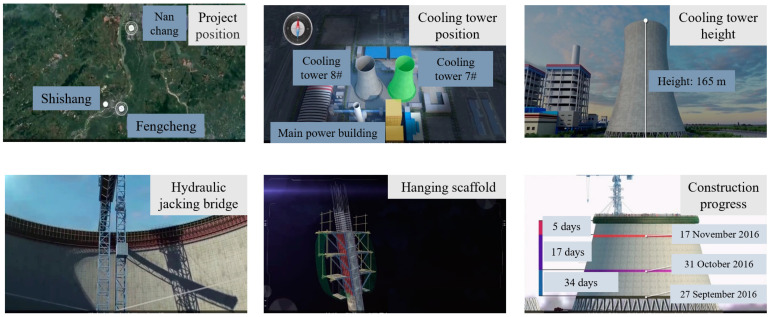
An overview of the cooling tower 7 project.

The Jiangxi Fengcheng Power Plant phase III expansion project belongs to the key power construction project of Jiangxi Province, with a total investment of 7.67 billion Yuan (about 1.2 billion US dollars). Cooling tower 7, which was 165 m tall and was made of reinforced concrete, was located northeast of the main plant. The construction of the drum wall adopted the hanging scaffold turning over the process, and a hydraulic jacking bridge was arranged inside the tower. The construction of cooling tower 7 started on 11 April 2016. By the time of the accident, the 52nd section of the tube wall concrete was poured to a height of 76.7 m. An overview of the project is shown in Figure 2.

With coal prices soaring and local governments pressing for political achievements, the construction team wanted the project to be in operation as soon as possible. Thus, the construction company proposed “a plan to reach the top by the end of the year”. The project department of the general contractor would have submitted the “Matters concerning Milestone” plan to the construction and supervision units, and the project duration of the construction unit would be adjusted from 212 days to 110 days, reducing the construction period by 102 days. After adjusting the construction period, the project department of the construction unit, the supervision unit, and the general contracting unit did not evaluate the shortened construction period, nor did they present the corresponding construction organization and safety measures. That is, participants did not have enough potential safety risk expectation/anticipation.

**Figure 3:**
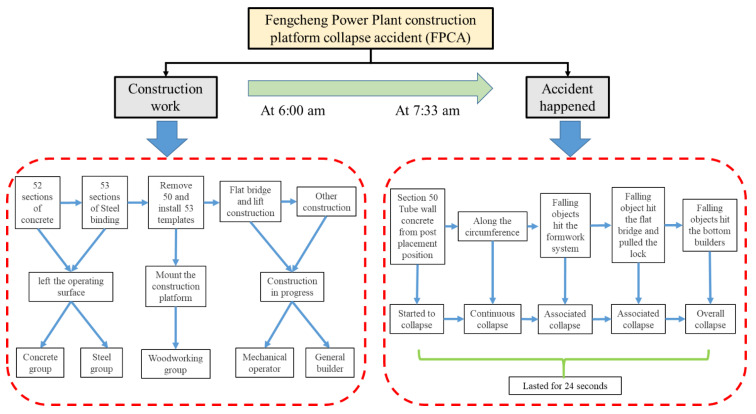
Overview of the FPCA.

The full incident occurred in two parts: (1) the construction operation period and (2) the accident per se (Figure 3). On 24 November 2016, at 6 a.m., 70 workers were distributed on the construction platform around the cylinder wall and 19 workers were working in the central shaft at the bottom of the cooling tower and the pool. At 7:33 a.m., section 50, the concrete of the drum wall of the cooling tower 7, began to collapse at the point where the concrete pouring was completed. During this period, the falling object hit the attached zipper of the flat bridge, thereby causing the bridge to shake and collapse. The accident lasted 24 s.

### 2.2. Hybrid Approach Designed for the Accident Analysis

By estimating construction safety risks based on complicated causal mechanisms analysis, occurrence mechanisms of the accident can be explained. FPCA was a complex and nasty collapse accident, involving not only technical factors, but also management factors. In this research, a hybrid approach integrating ETM and SD was proposed to delineate the entire evolution stage of the construction accident and estimate the involved safety risks. The ETM can be used to explain the gradual process of the change of dangerous energy transfer from the microscopic energy perspective, grasping the internal essential characteristics of the accident. Meanwhile, the SD model can be used to analyze multiple influence relations among participants of project management from the perspective of macro management, explaining the external characteristics of the accident. By incorporating technology- and management-related factors, the integration of ETM and SD model is effective in understanding the complexity of the entire accident and the blockage and control measures.

**Figure 4:**
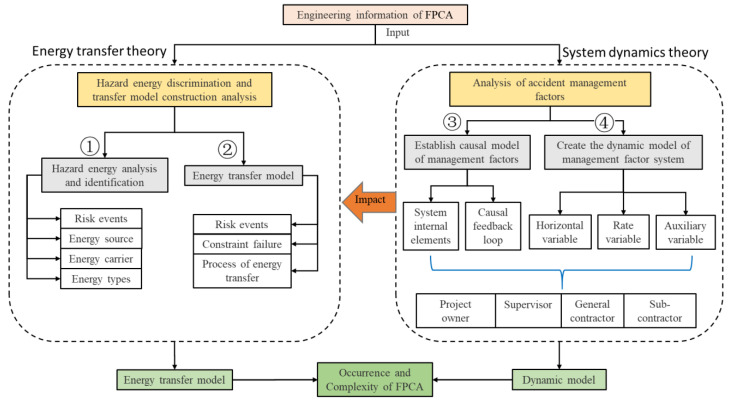
The hybrid approach for accident analysis of the FPCA.

In this research, the accident analysis framework system based on the ETM and SD theory integration methods is shown in Figure 4. Poor management is the subjective human factor that drives changes in energy transfer, while the energy transfer changes are the objective essential causes of accidents. The main steps of this research are shown, as follows.

Step 1. Collection of reading materials, with the accident investigation report of the FPCA as the main source of information; addition of the information about the occurrence and evolution of the accident as the auxiliary data.

Step 2. Establishment of the ETM according to the determination of engineering risk events, identification of hazardous energy, and constraint failure factors.

The ETM mainly includes two parts: the identification of dangerous energy and the establishment of the ETM per se. First, the dangerous energy steps are identified: (a) risk events are established; (b) energy sources and energy delivery to carriers are analyzed; and (c) energy types (N_*j*_) are evaluated. Then, an ETM is established, which mainly consists of the analysis of the failure factors of the energy constraint and the change in the energy transfer (Equations (1)–(3)).

T*_i_* = T_0_ + T*_i_*(N*_j_*), *i* = 1, 2, 3,…; *j* = 1, 2, 3, (1)

T*_i_*(N*_j_*) = *f*_(*i*−1)_(N*_j_*) + *f_i_*(N*_j_*) + *f*_(*i*+1)_(N*_j_*), *i* = 1, 2, 3,…; *j* = 1, 2, 3,… (2)

*f* = {E*p*, E*k*,…} (3)

In the above, T*_i_* is the energy transfer of ontology *i*, N*_j_* is the type of energy *j*, and *f_i_*(N*_j_*) is the calculation method for different energy types. E*p* is the geopotential energy, and E*k* is the kinetic energy.

Step 3. The SD model of accident management factors is constructed by analyzing the causal relationship between the accidents and the complex relationship between the participants in the construction of engineering projects.

The causal model of accident management factors was first established from the perspective of the construction project participants: (a) the internal elements of the system were analyzed; (b) the causal feedback loop was established, and then a dynamic model of the management factor system was created, including three analysis variables, namely horizontal, rate, and auxiliary variables. A thorough analysis of complex management factors is the most effective way to prevent and stop accidents.

Step 4. The ETM and the accident management factor SD model are integrated for analyzing the fundamental factors that lead to the occurrence of accidents comprehensively and for integrating the technical and management factors to explain the complexity of accidents more clearly, thereby providing a beneficial reference for the blockade and control of the accidents’ occurrence.

## 3. Collapse Accident Analysis of the Construction Platform Based on the ETM

### 3.1. Identification and Analysis of Hazardous Energy

The energy sources of the accident must be determined. Energy can be a segment, a place, a piece of equipment, or a situation with dormant energy or materials that carry the risk of injury, death, property loss, and environmental damage, such as moving parts of machinery, explosive materials, and moving vehicles. Energy sources for different types of accidents vary and the seven sub-item risk events in the production of the Fengcheng power plant in Jiangxi Province in Table 2. The sources and types of dangerous energy in the project are listed in Table 3. Engineering materials and loads are the main energy sources, and gravitational potential energy is the main dangerous energy source.

### 3.2. Analysis of Changes in the Energy Transfer

**Figure 5:**
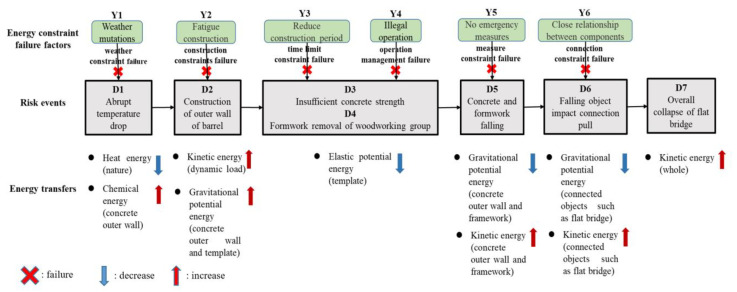
Evolution model of the energy constraint failure analysis for the FPCA.

The theory of energy transfer discusses the mechanism of accidents caused by energy transfer in engineering from the perspectives of physics, engineering, and management. Changes in the transfer of dangerous energy are considered to underlie accidents; consequently, restricting the transfer of dangerous energy is the most effective way to prevent accidents. Therefore, by establishing the evolution model of the energy constraint failure analysis and the evolution model of the energy transfer change, the process of the horizontal energy transfer change was analyzed in detail (Figure 5). The analysis of this model demonstrated that only by restraining and blocking the generation and transfer of dangerous energy it is possible to effectively control and reduce the occurrence of serious safety accidents. The FPCA had seven risk events (D1–D7) and six energy constraint failure factors (Y1–Y6). The detailed energy analysis process of this event is shown as follows.

1. The natural heat energy affected the release of chemical energy on the outer wall of the cylinder, owing to the abrupt temperature drop (Y1: weather constraint failure).
2. The amount of the stored energy increased rapidly and the energy transformed into the kinetic energy of concrete, owing to the construction of the outer wall of the cylinder (Y2: construction constraints failure) and the gravitational potential energy of the outer wall and the formwork itself.
3. Based on this kinetic energy, owing to the carpentry group’s illegal mold removal (Y3: time limit constraint failure) and the scaffold’s gravitational potential energy, the kinetic energy continued to increase substantially.
4. At the same time, the removal of the supporting formwork was out of order (Y4: operation management failure). The elastic potential energy of the supporting formwork was relieved, which further weakened the resistance to the gravitational potential energy of the concrete outer wall.
5. The concrete wall began to collapse with gravitational potential energy rapidly transforming into dynamic potential energy. Then, more objects, such as attached formworks, fell (Y5: measure constraint failure).
6. Finally, owing to the close connection between components of construction platform and the cylinder wall (Y6: connection constraint failure), falling objects impacted the construction platform. Connected lasso rods of the flat bridge were also damaged, thereby causing the tragic collapse, with the gravity of potential energy transforming into kinetic energy.

Under the six energy constraint failure factors mentioned above, the hazardous energy involved in seven risk events was unrestrained. That is, the energy transformation process was out of control. The hazardous energy (e.g., chemical energy, gravitational potential energy, and kinetic energy) was released completely along the connecting path of the structural body.

## 4. Analysis of the Accident Management Factors Based on the SD Theory

### 4.1. The Causal Loop Diagram of Management Factors in the Collapse Accident

**Figure 6:**
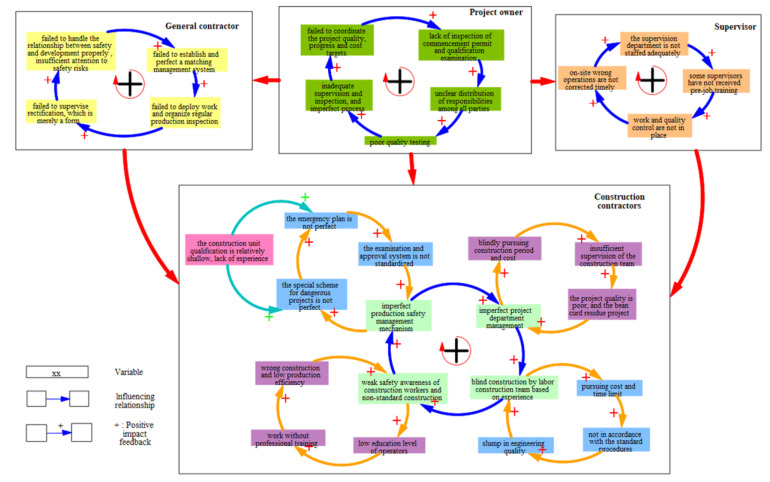
Causal loop diagram of participant management factors.

The analysis of the relationship between the construction safety accidents and engineering project management is complex and systematic, and depends on various inter-related factors [21]. According to the FPCA investigation report, the causal role of the management factors, associated with the project owner, the supervisor, the general contractor, and the construction contractor, was first considered for determining the system boundaries (Figure 6).

As shown in Figure 6, management factors within and between parties and their evolutionary paths (i.e., feedback loops) leading to the accident were summarized and expressed. That is, the causal model of management factors for all parties determined the influencing relationships between different system components. In particular, the main causal feedbacks of management factors within four parties were positive. Based on the system boundaries’ determination above, the causal relationships between the management elements of each party can be further analyzed quantitatively using the SD model.

### 4.2. The System Dynamic Model of the Management Factor System for Safety Risk Estimation

**Figure 7:**
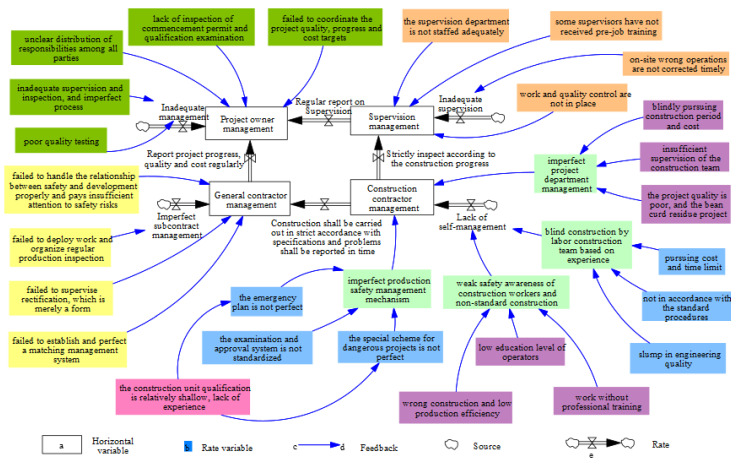
Dynamic evolution of the management factor system in the FPCA.

Based on the causal analysis of the FPCA and the investigation report of the accident, the SD model of the accident management factors was established using the system dynamics software Vensim (Figure 7).

As shown in Figure 7, more intuitive symbols have been used to describe the logical relations between system elements and to clarify the feedback and control styles of the system. Different colors were used to distinguish management factors of different horizontal variables. From the participants’ perspective, by analyzing the causal loop diagram, the model presents that the horizontal variables in the entire system include the management of the project owner, the supervisor, the general contractor, and the construction party (sub-contractor). Rate variables include insufficient management, insufficient regular reporting and monitoring, imperfect subcontracting management, and lack of self-management, etc. The remaining variables are the auxiliary variables (constants). Further, based on the SD model with different types of variables distinguished, the establishment of mathematical relations between various variables for computer simulation can be supported. In this way, a more quantitative and accurate analysis can be achieved. Accident management factors, based on the main dynamic evolutions of the management factor system, are analyzed as follows.

1. From the construction party (sub-contractor) perspective, wrong construction, low production efficiency, low level of education of the operators, and lack of professional training for the job resulted in the weak safety awareness of the operators, which was not in accord with construction standards. Construction quality plummets did not follow standard procedures, and the pursuit of cost and time limits led to the labor construction team’s numb construction experience, thereby resulting in the lack of self-management of the construction site. Relatively shallow qualifications and insufficient experience in construction units led to imperfect emergency plans and special plans for dangerous and large projects, as well as a nonstandard examination and approval system, which led to imperfect production safety management mechanisms. The blind pursuit of construction costs, inadequate supervision of the construction team, and poor project quality led to imperfect project department management.
2. From the point of view of the general contractor, poor work deployment and lack of regular production inspection led to imperfect subcontracting management. Failure to properly manage the relationship between safety and development, insufficient attention to safety risks, ineffective supervision and rectification, mere formality, and failure to establish and perfect a matching management system led to the poor management of the general contractor.
3. From the supervisor’s point of view, there was a failure to correct the wrong operation on-site in time. The supervisor’s supervision was insufficient. The supervision department was not equipped with sufficient personnel, some supervisors have not received induction training, and work and quality control standards were not in place, thereby resulting in insufficient supervisor management.
4. From the perspective of the project owner, the supervision and inspection were not in place, the process was imperfect, and quality detection was not in place, thereby leading to mismanagement. The contract, signing of the project contracting unit, inspection of the permit to start the work, and qualification examination of the construction unit, failed to coordinate the quality, progress, and cost of the project. The unclear distribution of responsibilities among all parties led to the lack of management of the construction party.
5. From the perspective of the overall system, the construction unit lacked self-management, did not strictly follow construction standards, and did not report to the general contractor and the supervision unit in time. The supervision unit did not strictly control the construction progress and quality, thus leading to a serious deficiency in the management of the general contractor and the supervision unit. The general contractor failed to report the progress of the project, quality, and cost to the project owner unit regularly. Moreover, the supervision unit failed to report regular supervision of the construction unit to the construction unit, ultimately leading to a serious deficiency in the management of the project owner unit.

## 5. Results and Discussion

For improving the safety management level of construction projects, a novel and integrated approach were proposed in this research to delineate the entire evolution stage of the construction accident. Specifically, the ETM and SD theories were adopted to estimate the involved construction safety risks with complicated causal mechanisms analyzed, from the perspectives of technology and management.

### 5.1. Safety Risk Estimation of Construction Project from the Perspective of Technology

In this research, the mechanism of the accident was first analyzed from the perspective of the energy constraints’ failure and control based on the ETM. Safety risk estimation was conducted focusing on the entire evolution stage of the construction accident. The seven event risk sources associated with energy were explored, including energy production, storage, and transfer, as well as the types of energy, among which the gravitational potential energy and the chemical energy were the main sources of dangerous energy in the accident. The constraint failure led to the transfer of the gravitational potential and chemical energies to the kinetic energy, and the failure of the kinetic energy constraint became the direct cause of the collapse accident. Injuries are usually caused by external forces that last longer than the body can handle in accidents. Work is the product of forces and distances. The dangerous energy can be transferred and changed in the construction body, which is a novel angle of view to analyze accidents. The FPCA is a typical accident caused by energy transfer among formwork, concrete, platform, tower crane, and workers. The analysis of FPCA based on ETM indicated that the platform collapse accident was a considerable engineering accident caused by the failure of a series of energy constraints and the transfer of dangerous energy.

### 5.2. Accident Network from the Perspective of Management

**Figure 8:**
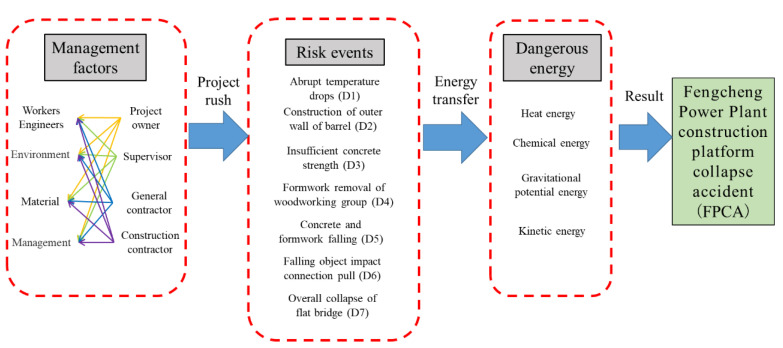
Flowchart of the project management and energy transfer changes.

After understanding the external energy transfer process, the coupling of dynamic management factors and worker unsafe behavior in the complex collapse accident was then analyzed. To further understand the dynamic and complex characteristics of FPCA, the internal system structure based on SD was analyzed for the management factors among the participants. The analysis demonstrated that this safety accident occurred not only owing to technical factors but also owing to management factors, which were closely related to each other (Figure 8). Multiple relationships among complex management factors and the influence of energy transfer constituted a complex accident-related network. Significantly, the energy transfer process is mainly due to situations created by the behavior of man. Based on Figure 8, some of the key management factors of the FPCA were discussed as follows.

1. Based on the analysis results, it suggests that the management factor was the key node that led to the accident in the accident management factor SD model. Owing to the lack of the management experience of the project owner, the supervisor, the general contractor, the construction contractor, and other participants, the slogan “100 days of construction” was presented to reduce the construction time. To meet the requirements of the claimed construction time, the construction party disassembled the molds in violation of regulations, thereby resulting in the occurrence of risk events, such as insufficient concrete strength and disassembled molds.
2. After the adjustment of the construction period, no construction party has demonstrated and evaluated the shortened construction period. Thus, neither corresponding construction organization measure nor security measure has been put forward. In this case, the subsequent construction activities and construction process management were conducted under an invalid construction plan. Further, ignorance of measures on sudden drops in air temperature and unsupervised construction activities contributed to the accident.
3. The construction contractor (i.e., sub-contractor) was the object that should have received attention during the construction, the key node in the accident-related network, and the key activity that corresponded to the generation and transfer of dangerous energy. The corresponding management countermeasures focused on the management and control of this link, which could reduce the connectivity of the accident-related network, could effectively reduce the efficiency of accident transmission, and so could reduce the probability and severity of such engineering accidents.
4. The quality of the workforce is another key management factor of the FPCA. In this project, to reduce the management cost of permanent worker groups, the labor subcontractor illegally lent the qualification and recruited many social natural persons as a labor force, without actual management. Only a small number of professional construction technical personnel had been arranged. The professional quality of the working group was low, and efficient management was difficult. Before the accident, the surface concrete solidified but did not reached sufficient strength. However, under the order to remove the supporting formwork, workers without construction experience executed it, and then the accident happened.

After the occurrence of risk events, energy transfer started, thereby converting ordinary energy into dangerous energy. The overall amount of dangerous energy increased dramatically. Quantitative changes caused qualitative changes, ultimately leading to the occurrence of the accident.

### 5.3. Management Implications Based on the Hybrid Accident Analysis Approach

Stricter oversight of management problems could have effectively reduced the occurrence probability of this accident. All project participants should have risk awareness and pay enough attention to potential safety risks of changes in the project plan. Correspondingly, timely and comprehensive construction organization and safety measures were needed.

The construction unit, as the first manager of the site, was also the main person in charge of the party responsible for the accident. The lack of self-management in the construction unit was the root cause of the accident. Only by strengthening the safety management of the construction site and strictly following the construction standard could the probability of the accident be effectively reduced.

The project owner unit was the key to controlling the quality of the project as the overall controller of the entire life cycle of the project. The project owner should have clarified the responsibilities of each subject to the supervisor, the general contractor, and the construction party, and should have supervised each sub-party in strict accordance with the procedures rather than in mere formality.

## 6. Conclusions 

This study is an important investigation on the evaluation of safety risks of construction projects, by delineating the entire evolution stage of a construction accident. Specifically, a novel approach and comprehensive analysis method were proposed. The mechanism of the FPCA was systematically analyzed based on the ETM from the technological perspective. The SD theory was used for constructing the network diagram of the accident management factors and for analyzing the network structure, to explore and understand the change processes, essential characteristics, and the complex characteristics of accident management from the management level. This research demonstrated that the collapse accident was essentially associated with the transfer of dangerous energy, caused by the failure of a series of energy constraints. Thus, the construction platform’s gravitational potential energy and chemical energy were converted into kinetic energy after the failure of the construction constraints. Moreover, the failure of the kinetic energy constraint led to the collapse accident. The analysis of the network structure of management factors before the accident further verified that the aforementioned project management was the key to the project’s safety control. Strengthening supervision and management and blocking the transfer of dangerous energy could have effectively reduced the connectivity of the accident-related network. It is expected to provide a useful reference for understanding the processes underlying the occurrence, risk management, and prevention of engineering accidents.

There are two limitations in this study. First, dangerous energy changes occurred with the work activities, therefore, future studies should identify the relative hazards and take safety measures to prevent different energy transformations. Another limitation is that the management factors coupled with the dangerous energy exacerbate the accident, while training or information technologies are being used in risk management for engineers. Future research studies should conduct new training or models to enhance the compliance and reliability of construction workers and engineers.

## Figures and Tables

**Table 1:** Main investigations on risk management and accident analysis in engineering construction.

No.	Method	Application Examples
1	Deep learning	Computer vision for identifying unsafe behavior and work conditions [4] Identification of the most dangerous risk combinations [5] Examination of the relationship between causal variables [8] Discussion on the complexity of subway construction accident networks [9]
2	Building information modeling (BIM)	A BIM-based hazard recognition and evaluation methodology [7]
3	Computer simulation	Examination of the relationships between different safety leaderships [10]
4	Systems science	Knowledge dynamics-integrated map [6] Interpretative structural model [11] Accident causality theory [12]
5	Energy transfer theory	Identifying energy sources and analyzing invalidation of energy restraints in accidents [13]
6	System dynamics (SD)	Analysis of intervention strategies for unsafe behaviors [14]

**Table 2:** Analysis of phased risk events in construction.

Phase	Process Description	Risk Events
The first stage	In the case of an abrupt temperature drop, the construction unit rushed to complete the task, resulting in the lack of strength of the section 50 concrete of cooling tower No. 7.	Abrupt temperature drop (D1); Construction of outer wall of barrel (D2); insufficient concrete strength (D3)
The second stage	The construction adopts the hanging scaffold formwork turning over the process. The labor team decides to dismantle 50 formworks by themselves, resulting in the continuous collapse of the concrete and formwork system of more than 50 cylinder walls.	Formwork removal of woodworking group (D4); concrete and formwork falling (D5)
The third stage	The falling object impinges on the attached zipper of the flat bridge connected with the inside of the cylinder wall, causing the whole flat bridge to collapse.	Falling object impact connection pull (D6); overall collapse of flat bridge (D7)

**Table 3:** Source and type discrimination of dangerous energy in construction.

Risk Events	Production and Storage of Energy	Energy Source	Energy Types
Abrupt temperature drops (D1)	Nature, concrete	Temperature	Heat energy, chemical energy
Construction of outer wall of barrel (D2)	Concrete, formwork	Material, load	Gravitational potential energy, kinetic energy
Insufficient concrete strength (D3) and formwork removal of woodworking group (D4)	Formwork, concrete	Material, strength	Elastic potential energy
Concrete and formwork falling (D5)	Concrete, formwork, scaffolding	Material, load	Gravitational potential energy, kinetic energy
Falling object impact connection pull (D6)	Falling objects, zips	Object, load	Gravitational potential energy, kinetic energy
Overall collapse of flat bridge (D7)	Flat bridge	Object, load	Kinetic energy

## Data Availability

All data related to this study is explicitly plotted in the figures in this article.
